# A Large Outbreak of Legionnaires’ Disease at a Flower Show, the Netherlands, 1999

**DOI:** 10.3201/eid0801.010176

**Published:** 2002-01

**Authors:** Jeroen W. Den Boer, Ed P.F. Yzerman, Joop Schellekens, Kamilla D. Lettinga, Hendriek C. Boshuizen, Jim E. Van Steenbergen, Arnold Bosman, Susan Van den Hof, Hans A. Van Vliet, Marcel F. Peeters, Ruud J. Van Ketel, Peter Speelman, Jacob L. Kool, Marina A.E. Conyn Van Spaendonck

**Affiliations:** * National Institute of Public Health and the Environment, Bilthoven, the Netherlands; † Municipal Health Service Zuid-Kennemerland, Haarlem, the Netherlands; ‡ Regional Laboratory of Public Health Haarlem, Haarlem, the Netherlands; § Academic Medical Center, Amsterdam, the Netherlands;; ¶ National Outbreak Structure for Infectious Diseases, Den Haag, the Netherlands; # Regional Laboratory of Public Health Tilburg, Tilburg, the Netherlands

**Keywords:** Legionnaires’ disease, outbreaks, environmental microbiology, pulsed-field gel electrophoresis, case-control studies, cohort studies, seroepidemiologic studies

## Abstract

In 1999, an outbreak of Legionnaires’ disease affected many visitors to a flower show in the Netherlands. To identify the source of the outbreak, we performed an environmental investigation, as well as a case-control study among visitors and a serologic cohort study among exhibitors to measure exposure to possible sources. Of 77,061 visitors, 188 became ill (133 confirmed and 55 probable cases), for an attack rate of 0.23% for visitors and 0.61% for exhibitors. Two whirlpool spas in halls 3 and 4 of the exhibition and a sprinkler in hall 8 were culture positive for *Legionella pneumophila*. One of three genotypes found in both whirlpool spas was identical to the isolates from 28 of 29 culture-positive patients. Persons who paused at the whirlpool spa in hall 3 were at increased risk for becoming ill. This study illustrates that whirlpool spas may be an important health hazard if disinfection fails.

From March 7 to 11, 1999, 10 patients with severe pneumonia were admitted to a hospital in Hoorn, in the northern part of the Netherlands. The clinical condition of these patients deteriorated quickly and unexpectedly, with eight requiring mechanical ventilation. On March 11, six of the eight patients were diagnosed with Legionnaires’ disease on the basis of a positive *Legionella* urine antigen test. Additional patients with severe pneumonia were sent to another hospital since all the respirators at the Hoorn hospital were in use. Two of these patients were subsequently diagnosed with Legionnaires’ disease by urine antigen test.

An exploratory case-control study using a questionnaire on exposure to potential sources was conducted; six confirmed and four probable cases were included. All patients and 3 of 21 controls had visited the West Frisian Flower Show (p<0.001). Since no other environmental risk factor was identified, the outbreak investigation focused on the flower show. This show is an annual event that includes an agricultural and consumer products exhibition, located in the nearby town of Bovenkarspel. Held from February 19 to 28, the flower show attracted 77,061 visitors. After a nationwide warning was issued to ensure detection and appropriate treatment of any additional cases, it became clear that >180 persons had been affected. We report how the source of the outbreak was identified by a combination of an environmental investigation, a case-control study among flower show visitors, and a cohort study among >700 exhibitors.

## Methods

A confirmed case of Legionnaires’ disease was defined as radiologically confirmed pneumonia in a visitor to the exhibition or a member of the exhibition staff, with onset no earlier than February 19, 1999, and no later than March 21, 1999, as well as laboratory evidence of *Legionella pneumophila* infection. Laboratory evidence included isolation of *L. pneumophila* from respiratory secretions, detection of *L. pneumophila* antigens in urine, or a fourfold or higher rise in antibody titers to *L. pneumophila* in paired acute- and convalescent-phase sera, as reported by clinicians. A probable case was defined as radiologically confirmed pneumonia with onset no earlier than February 19, 1999, and no later than March 21, 1999, in an exhibition visitor or a member of the exhibition staff who did not meet laboratory criteria for a confirmed case, but who had no laboratory evidence of infection by other microorganisms. All local health services and hospitals in the Netherlands were informed of these criteria and asked to report cases of pneumonia in persons who had visited the exhibition. Unsolicited case reports from the public were also recorded. Finally, all Dutch clinical medical microbiology laboratories were asked to send clinical *Legionella* isolates from flower show-related Legionnaires’ disease patients to the National Institute of Public Health and the Environment (RIVM) for serotyping and genotyping.

### Environmental Risk Assessment

A map of the water system at the exhibition site was made to facilitate visual inspection for circulatory dead ends and other potential locations of stagnant water. In addition, we interviewed all exhibitors to compile an inventory of all products using water that had been displayed at the exhibition. Based on these interviews, an 8-point risk-assessment scale was developed to discriminate among the products used during the exhibition. For each of the following items, 1 point was given: use of water; use of water at 20°C to 43°C (the temperature range within which *Legionella* can amplify to dangerous concentrations) (*1*,*2*); use of water at 37°C (the optimal temperature for *Legionella* growth) (*3*); no disinfection of water at 20° to 43°C; no changing of water at 20° to 43°C; occasional misting of water at <60°C; continuous misting of water at <60°C; and substantial surface for misting of water at <60°C.

Two weeks after the end of the exhibition, we began to obtain water and swab samples from all potential sources of *Legionella*. The water samples were concentrated by membrane filtration (0.2 μm), and filtered residues were resuspended in 1 mL sterile water. Of this suspension, 100-μL samples were cultured without dilution and after 10- and 100-fold dilutions on buffered charcoal yeast extract agar with alpha-ketoglutarate (BCYE-alpha) and a selective supplement with dyes and the antibiotics polymyxin, anisomycin, and vancomycin (Legionella MWY Selective Supplement SR118, Oxoid Ltd., Hampshire, England). Plates were incubated at 37°C with increased humidity. In case of bacterial overgrowth, cultures were repeated after pretreatment by heating 30 minutes at 50°C. Swab samples were dispersed by immersion in 1 mL sterile water and cultured as described. Cultures were examined microscopically daily for 14 days. In case of persistent overgrowth, ceftazidime was added to the media. Colonies suspected of being *Legionella* were subcultured to blood agar and BCYE-alpha agar. Identification was confirmed by biochemical tests. *Legionella* isolates were serogrouped by using commercial kits containing antisera against *L. pneumophila* serogroups 1-14, *L. longbeachae* 1 and 2, *L. bozemanii* 1 and 2, *L. dumoffi*, *L. gormanii*, *L. jordanis, L. micdadei*, and *L. anisa* (*Legionella* Latex Test, Oxoid Limited, Hampshire, England; *Legionella* antisera “Seiken,” Denka Seiken Co. Ltd., Tokyo, Japan). Isolates were genotyped by pulsed-field gel electrophoresis (*4*) and amplified fragment-length polymorphism (*5*).

### Case-Control Study

To measure visitor exposure to possible sources of *Legionella*, we used a questionnaire, a set of situational drawings, and a floor plan of the exhibition site. The questionnaire addressed health status and details about visits to different parts of the exhibition or displays of devices capable of spreading *Legionella* (*6*–*8*). As controls, a random sample of 2,500 men and 2,500 women born before 1960 were selected from the municipal population register. A request for participation in the study was sent to all these persons, but they were asked to reply only if they had visited the exhibition. Of the first 469 who replied, all 196 men and a random selection of 203 women were sent the questionnaire. Respondents were excluded as controls if they had symptoms of respiratory infection within 20 days of their exhibition visit (for pneumonia and bronchitis) or within 4 days of their visit (for influenzalike illness). Both cases and controls were asked to indicate the route they had walked and the stands they had visited on a floor plan of the exhibition site and situational drawings of stands at which devices using water were displayed. To avoid bias, drawings of stands at which products were demonstrated that did not use water were also included. Ill persons were interviewed personally or by proxy.

Variables that were significant in univariate analysis were entered in a multiple logistic regression model. With the use of backward elimination, independent predictors of becoming ill, adjusted for age and sex, were established. Variables were retained in the model if the likelihood ratio test was significant (p<0.1).

### Cohort Study

Letters were sent to exhibition volunteers, staff of the company organizing the exhibition, and all exhibitors (n=1,616) (with the exception of persons with confirmed and probable cases), asking them to complete a questionnaire regarding their health status before and after the exhibition. Questions were included about principal work location during the exhibition. Participants were asked to have paired blood samples drawn and sent to RIVM. The first samples were taken by the end of March and the second by mid-May. Serum immunoglobulin (Ig) M and IgG antibodies against *L. pneumophila* were detected by indirect enzyme-linked immunosorbent assay (Virion-Serion, Würzburg, Germany). For every 63-cm^2^ area of the exhibition site, the geometric mean IgM and IgG titers of the nearest 35 respondents were determined and plotted after smoothing by using the highest titer in paired sera for each respondent. The correlation of the logs of the antibody titers and distance to aerosol-generating devices was analyzed by linear regression, after the data were adjusted for age and sex of respondents, smoking status, underlying disease, and time worked in each hall. In all analyses, data were included only from persons who had worked at the exhibition after February 22.

## Results

**Figure 1:**
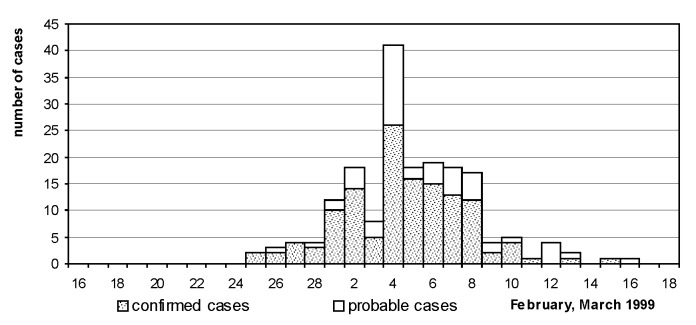
Dates of onset of illness in 186 cases of Legionnaires’ disease, February 16-March 18, 1999.

A total of 188 cases of Legionnaires’ disease (133 confirmed, 55 probable) were reported in visitors (178) and exhibitors (*9*), originating from throughout the Netherlands. An additional 21 patients had physician-diagnosed pneumonia but no radiologic studies, and 9 patients had positive urine antigen tests but insufficient clinical data. Dates of onset ranged from February 25 to March 16 (Figure 1). The median age of patients was 66 years (range 20 to 91 years), and the male:female ratio was 1:4. Diagnosis was confirmed by culture in 29 cases, urine antigen test in 100 cases, and serologic testing in 53 cases (Table 1). In 54 cases, two or more tests were positive. Among patients for whom data were available and who visited the exhibition once (n=136), the reported incubation period was 2 to 19 days (median 7 days); in 22 cases (16%) the time before onset of illness exceeded 10 days. All but one ill person had visited the exhibition after February 22. The exception was a 55-year-old woman with a history of chronic obstructive pulmonary disease and pneumonia who visited the exhibition on February 21. She visited hall 3 for 45 minutes and stopped near the whirlpool spa.

**Table 1:** Positive diagnostic tests results for 188 cases (133 confirmed and 55 probable) of Legionellosis, the Netherlands, 1999^a^

	Culture	Urinary antigen	Fourfold rise in titer	Direct immunofluorescence	PCR^b^	Single high titer
Culture	**29**	24	3	2	11	4
Urinary antigen	24	**100**	25	1	15	12
Fourfold rise in titer	3	25	**53**	0	1	0
Direct immunofluorescence	2	1	0	**2**	1	0
PCR	11	15	1	1	**18**	3
Single high titer	4	12	0	0	3	**18**

^a^The table is read as follows: of 29 patients who had positive cultures, 24 were positive by urinary antigen, 3 had a fourfold rise in titer, 2 were positive by direct immunofluorescence, and 11 by PCR, and 4 had single high titers.

^b^PCR = polymerase chain reaction.

**Figure 2:**
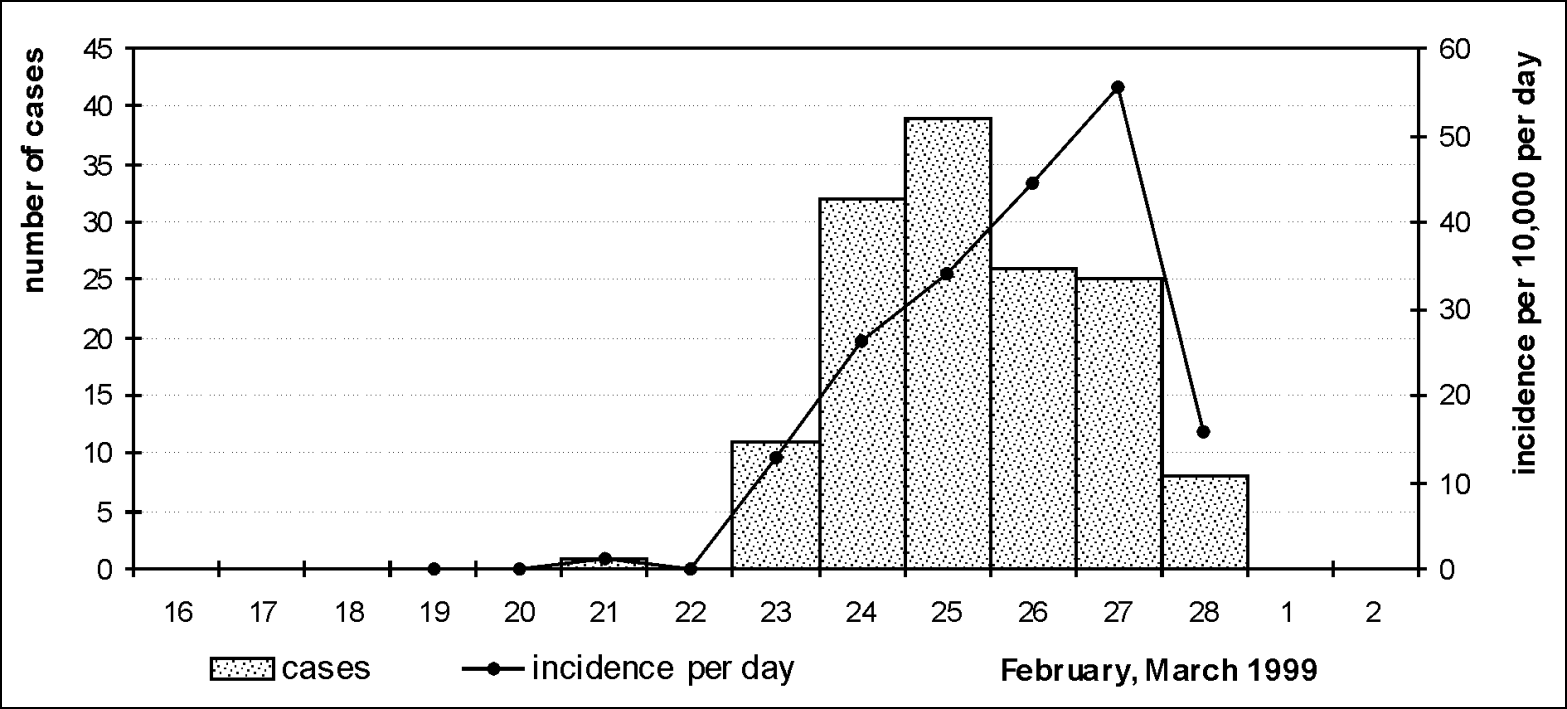
Confirmed and probable cases of Legionnaires’ disease per day of visit to flower show. Incidence per 10,000 visitors per day of visit, February 16-March 2, 1999.

**Figure 3:**
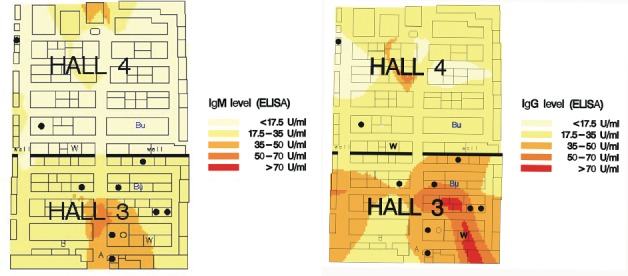
**a.** Smoothed mean geometric immunoglobulin (Ig) M antibody titers to *Legionella pneumophila* of nearest 35 exhibitors in hall 3 and 4 per 63 cm^2^ of exhibition area; confirmed and probable cases among exhibitors in halls 3 and 4. = confirmed case in exhibitor; = probable case in exhibitor; Bu = bubblemat; W = whirlpool spa. **b.** Smoothed mean geometric IgG antibody titers to *L. pneumophila* of 35 exhibitors nearest to whirlpool in halls 3 and 4 per 63 cm^2^ of exhibition area; exhibitors ill with confirmed and probable cases in halls 3 and 4. l = confirmed exhibitor case; = probable exhibitor case; Bu = bubblemat; W = whirlpool spa.

The attack rate for visitors was 178/77,061 or 0.23%. The daily attack rate increased from 0.011% on February 21 to 0.56% on February 27 (Figure 2). Ten of the exhibitors, volunteers, and employees became ill, for an attack rate of 0.61% among staff. Seven of these 10 staff members worked in the right side of hall 3 (Figures 3a and 3b). The attack rates for staff were 2.7% for hall 3 and 0.4% for hall 4 (Fisher exact test, p=0.005).

Of the 188 patients, 163 (87%) were hospitalized and 34 (21%) required mechanical ventilation. Seventeen persons with confirmed and 4 with probable cases died, for a case-fatality rate of 11%. The case-fatality rate was highest (17%) in patients >70 years of age.

Clinical isolates of 29 patients were available for genotyping. Twenty-eight were identical to the strain designated B-1, and one was identical to B-2.

### Environmental Risk Assessment

**Figure 4:**
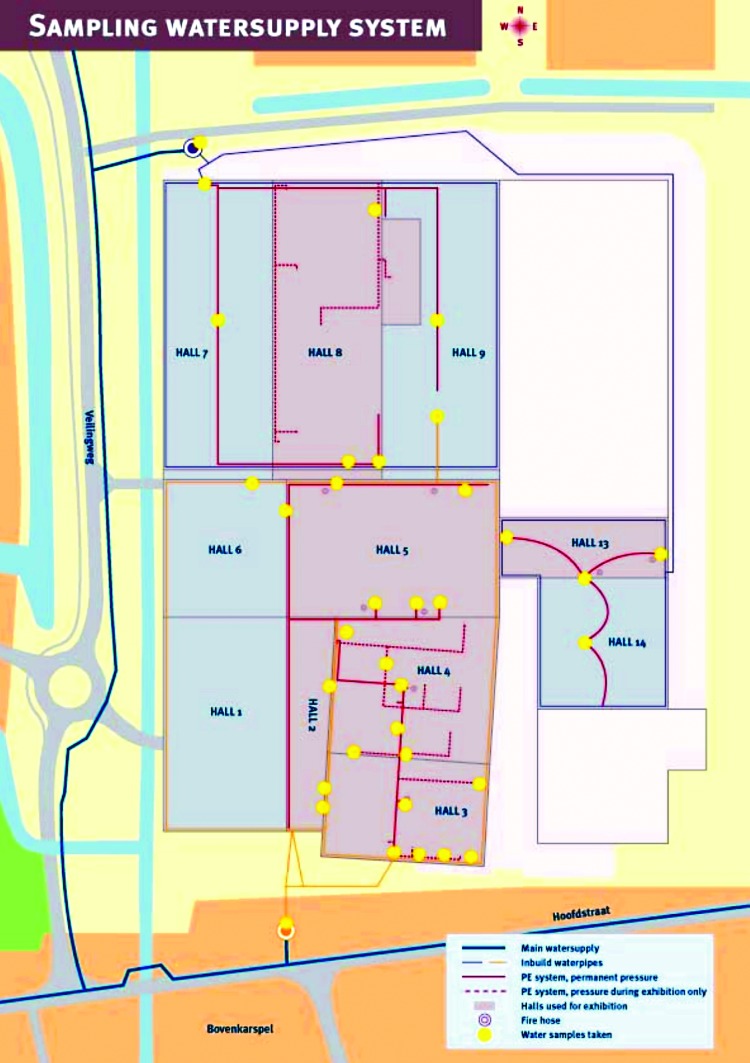
Water-supply system for exhibition hall, Bovenkarspel the Netherlands, 1999. PE = polyethylene.

The 11 halls of the exhibition site were supplied with water by two separate systems. The flower show was held in halls 2, 5, and 13; the consumer exhibition in halls 3 and 4; and the agricultural exhibition in halls 8 and 9 (Figure 4a). During the 3 months before the exhibition, the right side of hall 3 had been partitioned off to preserve flower bulbs at 30°C. In halls 5 and 13, 11 decorative fountains and a waterfall were installed. The temperature in these two halls was kept at ≤15°C to preserve the flowers. Our risk assessment of the water-using devices showed (Figure 4b) that a whirlpool spa in hall 3 posed the greatest risk (8 points), followed by a whirlpool spa in hall 4 (6 points), two bubblemats1 in halls 3 and 4 (4 points), 11 fountains in halls 5 and 13 (4 points), and a sprinkler installation in hall 8 (3 points). None of these devices were maintained with adequate disinfection. The whirlpool in hall 3 had never been used before, and its disinfection system failed. Ten samples were collected from the municipal water supply and 127 from the water system of the exhibition building (Figure 4a). Of 27 water-using devices that had been on display at the exhibition, 12 (including the whirlpool spas and the sprinkler installation) were available and still contained water (Figure 4c). A total of 145 samples were taken from these 12 devices. All cultures of specimens from the water supply system were negative for *L. pneumophila*, but the organism was cultured from paper filters of the whirlpool spa in hall 3 (>100 colonies), the whirlpool spa in hall 4 (2 colonies), and the sprinkler installation in hall 8 (15 colonies). Subsequent sampling of the inner tubing system of both whirlpool spas, 6 weeks after the exhibition ended, yielded abundant growth of *L. pneumophila* from swabs of the hall 3 whirlpool spa. No growth was found in the remaining water from the whirlpool spa in hall 4, which had a chlorine concentration of 0.64 mg/L.

**Figure 5:**
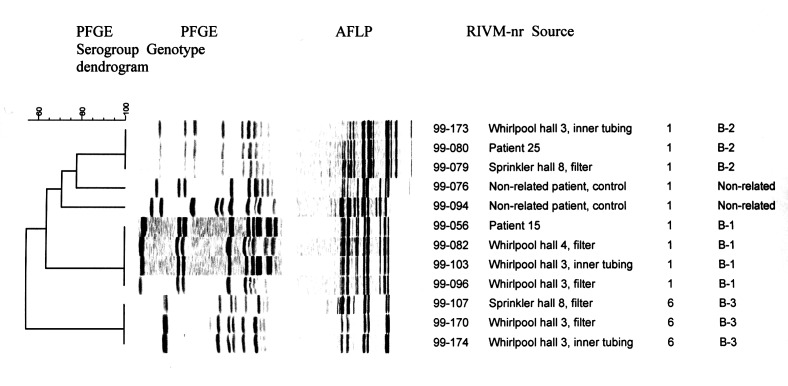
Pulsed-field gel electrophoresis (PFGE) and amplified fragment-length polymorphism (AFLP) patterns of a representative selection of clinical and environmental *Legionella pneumophila* isolates; the dendrogram shows clustering in PFGE. The AFLP and PFGE pattern of the isolate of patient 15 (genotype B-1) was found in 28 of the 29 isolates of culture-positive cases; the same pattern was found in isolates cultured from the whirlpool spas in halls 3 and 4. The AFLP and PFGE pattern of the isolate of patient 25 (genotype B-2) was unique among culture-positive cases; the same pattern was found in isolates cultured from the whirlpool spa in hall 3 and the sprinkler. ^1^A bubblemat is an inflatable rubber mat that causes a whirlpool-like effect when placed in a normal bathtub.

Serotyping and genotyping of environmental isolates yielded three distinct genotypes: two serogroup 1 isolates (called B-1 and B-2) and one serogroup 6 isolate (called B-3). Genotyping results for pulsed-field gel electrophoresis and amplified fragment-length polymorphism were in agreement (Figure 5). The filter and the inner tubing of the hall 3 whirlpool spa contained B-1, B-2, and B-3 (semiquantitatively B1>B2>B3), the filter of the hall 4 whirlpool spa contained B-1, and the filter of the sprinkling installation contained B-2 and B-3 (semiquantitatively B-2>B-3). Cultures of the inner tubing of the hall 3 whirlpool spa taken several months after the exhibition were still positive and yielded all three genotypes.

### Case-Control Study

The rates of response to the questionnaire and drawings were 85% and 58% for cases and 98% and 65% for controls, respectively. Thirty-six controls who reported symptoms of respiratory infection were excluded from the analysis. Analysis was restricted to data from 71 confirmed and 30 probable cases and 119 controls who visited the exhibition after February 22 (Table 2). The variables that remained in the multiple regression model were based on 62 confirmed and 21 probable cases and 105 controls (Table 3). Our analysis showed that smoking and length of stay at the exhibition were risk factors for infection. Length of stay exclusively at the consumer products exhibition showed an inverse relation, after data were adjusted for total length of stay. However, cases and controls on average spent the same amount of time at the consumer exhibition. Drinking tap water was not a risk factor. Visiting the whirlpool spa display in hall 3 was a risk factor. Visiting the bubblemat display in hall 3 was also associated with risk, but visiting a display of the same bubblemat in the gangway showed an inverse relation. Similar but not always statistically significant results were found when the analysis was limited to confirmed cases.

**Table 2:** Results of univariate analysis of data from questionnaires and drawings comparing host factors and visits with specific sites at the exhibition for cases and controls.

Study population	Respondents’ questionnaire and set of drawings; univariate OR^a^ (95% CI) (101 cases, 119 controls)	Respondents’ questionnaire and set of drawings; raw data (101 cases, 119 controls)
Age	1.1 (1.0 – 1.1)	na
Male	2.7 (1.6 – 4.7)	63/45
Underlying disease	7.2 (1.5 – 33.1)	37196
Smoking	2.0 (1.1 – 3.6)	42/31
Total hours at exhibition	1.7 (1.4 – 2.1)	36983
Hours at consumer exhibition	1.0 (0.8 – 1.3)	36891
Pausing at whirlpool spa in hall 3	4.2 (1.9 – 9.0)	41/21
Pausing at bubblemat in hall 3	3.7 (1.6 – 8.2)	37/17
Pausing in gangway of bubblemat in hall 3	0.4 (0.2 – 1.0)	24/35
Pausing at electric kettle in hall 3	3.0 (1.3 – 7.0)	26/12
Pausing at whirlpool in hall 4	2.4 (1.1 – 5.4)	31/20
Pausing at steam iron in hall 4	5.4 (1.4 – 22.0)	16/3

^a^OR = odds ratio; CI = 95% confidence intervals; na = not available.

**Table 3:** Logistic regression models of data from questionnaires and drawings comparing host factors and visits to specific sites at the exhibition for cases and controls

Study population	Respondents to questionnaire and set of drawings; OR^a^ (95% CI)(101 cases, 119 controls)
Age	1.1 (1.0 – 1.1)
Male sex	2.1 (1.0 – 4.5)
Smoking	6.0 (2.4 – 15.1)
Total hours at exhibition	2.2 (1.5 – 3.2)
Hours at consumer exhibition	0.5 (0.3 – 0.8)
Pausing at whirlpool spa in hall 3^b^	2.6 (1.1 – 6.6)
Pausing at bubblemat in hall 3^b^	3.0 (1.1 – 8.0)
Pausing in gangway of bubblemat in hall 3^b^	0.3 (0.1 – 0.8)

^a^OR = odds ratio; CI = 95% confidence interval.

^b^See Figure 3.

### Cohort Study

Of the exhibition staff, 880 responded to the cohort study questionnaire (54%), and 714 (44%) provided two analyzable blood samples. Geometric mean IgG and IgM titers were not associated with drinking tap water or contact with potting compost. Geometric mean IgM and IgG titers were significantly increased (p≤0.0002) among exhibitors in hall 3 but not among those in hall 4, compared with exhibitors working in other halls. Respondents who worked in the right side of hall 3 had the highest average antibody titers (Figures 3a and 3b). Multiple linear regression showed that proximity to both the whirlpool spa and the bubblemat in hall 3 was positively associated with increase in antibody titer, but no such relation was found for distance to the bubblemat and whirlpool spa in hall 4. Since the bubblemat and the whirlpool spa in hall 3 were situated close together, exposure to each was highly correlated and risk could not be differentiated. An inverse relation was found between the attack rate for staff with confirmed legionellosis and the distance of their workplace to the whirlpool in hall 3 (p=0.0009). Staff members who became ill and who worked in the right side of hall 3 differed from their colleagues in the same hall with respect to age, gender, and smoking habit (Table 4).

**Table 4:** Characteristics of persons with legionellosis who worked in the right half of hall 3 compared with staff members who did not get become ill and who worked in the same hall

	Cases in right half of hall 3 (n=7) n (%)	Cohort respondents in hall 3 (n=151) n (%)
Age group		
<30	0 (0)	23 (15.7)
30-39	0 (0)	24 (16.3)
40-49	1 (14)	29 (19.7)
50-59	4 (57)	46 (31.3)
60-69	2 (29)	20 (13.6)
≥70	0 (0)	5 (3.4)
Males	6 (86)	67 (45.3)
Smokers	4 (67)	44 (29.1)
Immunocompromised	0 (0)	5 (3.3)
History of pneumonia	1 (14)	5 (3.3)
History of diabetes	1 (14)	5 (3.3)

^a^Data missing for one case.

## Discussion

A new whirlpool spa, within 4 days after its installation, was the major source of one of the world’s largest outbreaks of Legionnaires’ disease. With 188 (133 laboratory-confirmed) cases, this outbreak is only exceeded by the original 1976 outbreak in Philadelphia (221 cases). Because our case definition for probable cases was broad, the 55 probable cases may include some persons with other, undetected causes of pneumonia. Detailed studies of clinical and laboratory-diagnostic characteristics of the patients are ongoing.

Despite these limitations, this outbreak of *Legionella* pneumonia is certainly the largest to be associated with a contaminated whirlpool spa. Although the overall attack rate (0.24%) was low for this outbreak in comparison with other nonhospital indoor outbreaks (4% to 7%) (*9*–*11*), the large number of visitors resulted in a large number of patients. The fact that the outbreak was not detected until 14 days after the first case of pneumonia was diagnosed, when 71 pneumonia patients had already been hospitalized, is remarkable. In hindsight, the first hospitalized patient could have been diagnosed on February 25, when only 30 to 35 of the eventual 188 patients had been infected. Although immediate diagnosis would have enhanced the possibility of timely public health intervention, the source of the outbreak is unlikely to have been discovered before the end of the exhibition. Late detection may be due partly to small-scale use of the *Legionella* urine antigen test in the Netherlands. Dutch physicians may have considered Legionnaires’ disease a rare event, since over the last 10 years no more than 45 cases per year have been reported and few community-acquired outbreaks have been described (*12*,*13*). In 2000, the number of reported cases in the Netherlands was 176, suggesting underdiagnosis in previous years.

No guidelines concerning the use and maintenance of whirlpool spa displays exist in the Netherlands. Our data demonstrate that contaminated spas may remain culture positive for months, perhaps as a result of stagnant water in their extensive inner tubing system. Because *Legionella* is ubiquitous in water systems, prevention of Legionnaires’ disease depends mainly on disinfection. This study shows that whirlpool spas may become a health hazard if their disinfection system fails. Bathing in whirlpool spas has led mainly to outbreaks of Pontiac fever (*14*–*18*) and, to lesser extent, of Legionnaires’ disease (*6*,*19*,*20*). Our data show that even staying in the vicinity of a whirlpool spa or walking in a hall where an operating whirlpool spa is on display may be important risk factors for Legionnaires’ disease. Considering the popularity of whirlpool spas at home and the number of exhibitions where they are displayed, we suspect that small outbreaks have occurred without detection.

Clearly, strict regulations concerning the use, maintenance, and display of whirlpool spas are needed. The public at large should be informed as to the potential health hazards posed by whirlpools spas in public facilities and at home.

Unique in this outbreak was the circumscribed time of exposure for each individual patient to an identified source of *Legionella* infection. The finding that in 16% of cases the reported incubation period exceeded 10 days has major clinical and public health consequences. This finding contrasts with that of the Philadelphia outbreak, when only two cases had such long incubation times (16 and 26 days, respectively) (*10*).

In this outbreak investigation, a unique combination of three epidemiologic approaches allowed a comprehensive understanding of the chain of events, even as the investigators were confronted with numerous potential sources, three of which were culture positive. Our simple risk assessment of devices capable of spreading *Legionella*-infected aerosols proved to be an effective and timely predictor of the likelihood that a device was a source of the outbreak. The assessment and subsequent cultures revealed that the whirlpool spa in hall 3 was most likely the major source because it had been in continuous operation and its water had not been changed during the exhibition, unlike the whirlpool spa in hall 4. The bubblemats in halls 3 and 4 were demonstrated in room-temperature water, which was changed several times during the exhibition. Both mats had been dried and stored by the time of the environmental sampling.

The results of the case-control study indicate that pausing at this whirlpool spa was the most important consumer-related risk factor. Information bias related to this outcome is probably minimal, since the Dutch news media never mentioned the site of the whirlpool spa when reporting on the origin of the outbreak.

The results of the cohort study show that the average antibody levels were highest in the right side of hall 3, near the whirlpool spa. Plotting the geometric mean IgM and IgG titers of the nearest 35 exhibitors per surface area demonstrated that proximity to the whirlpool spa in hall 3 was associated with an increase in antibody titers, whereas this association was absent in hall 4. These results correlate with the inverse relation between attack rate among staff members and distance to the whirlpool spa in hall 3. The smoothing technique used in our analysis gives an average antibody titer (for the nearest 35 exhibitors) per square meter. The exhibition hall was divided into 63-cm^2^ squares, and for each square the smoothed average antibody titer was calculated and a color was assigned corresponding to a certain titer range. The color pattern gives an idea of the pattern of infected aerosols or movement of exhibitors.

The whirlpool spa in hall 3 had just been purchased; it was filled on February 17 and kept at 37°C throughout the exhibition. The concentration of *L. pneumophila* must have risen to levels infectious for immunocompromised visitors from February 21 onward and healthy visitors from February 23 onward. Similar growth rates have been reported (*21*,*22*). The increasing attack rate per day indicates that the continued growth of *Legionella* led to spread of aerosols bearing ever-increasing infectious doses. The lower attack rate on the last day of the exhibition may reflect the different composition of the visitor population on Sundays, when young families with children predominated, compared with weekdays, when elderly visitors predominated.

Although all cultures of specimens from the two separate parts of the local water supply system were negative, it is probable but unproven that the *Legionella* strains cultured from the whirlpool spas and the sprinkler installation originated from the local system. The finding of identical genotypes in these devices supports this hypothesis.

In conclusion, this large, severe outbreak in the Netherlands shows that diagnosis of *Legionella* pneumonia should lead to prompt investigation of the source of infection. Our comprehensive epidemiologic investigation identified a new whirlpool spa as the major source of the outbreak. Until strict regulations concerning the operation of whirlpool spas have been developed and issued, public exhibition of these devices in operation should be restricted.
