# Erratum Vol. 8, No. 1 

**DOI:** 10.3201/eid0802.020201

**Published:** 2002-02

In the article, "ALargeOutbreakofLegionnaires'DiseaseataFlowerShow,theNetherlands," by Jeroen W. Den Boer et al., errors appear in two figures and their legends. 

In **Figure 3,** part **3a** should be identified as IgM and part **3b** as IgG. The correct legends are as follows:

**Figure 3a.** Smoothed mean geometric immunoglobulin (Ig) M antibody titers to Legionella pneumophila of nearest 35 exhibitors in Halls 3 and 4 per 63 cm2 of exhibition area; confirmed and probable cases among exhibitors in halls 3 and 4. ● = confirmed case in exhibitor; ○ = probable case in exhibitor; Bu = bubblemat; W = whirlpool spa. 

**Figure 3b.** Smoothed mean geometric IgG antibody titers to L. pneumophila of 35 exhibitors nearest to whirlpool in halls 3 and 4 per 63 cm2 of exhibition area; exhibitors ill with confirmed and probable cases in halls 3 and 4. ●= confirmed case in exhibitor; ○ = probable case in exhibitor; Bu = bubblemat; W = whirlpool spa. 

In **Figure 4**, parts **b** and **c** were inadvertently omitted. The complete figure appears below. The correct legends are as follows:

**Figure 4. **Exhibition hall, West Frisian Flower Show, Bovenkarspel, the Netherlands, 1999. **a.** Circles indicate locations in water-supply system where water samples were taken. PE = polyethylene. **b.** Assessment of risk for Legionella pneumophila infection, by distance from water-using devices. **c.** Water samples taken and culture status, by distance from water-using devices.

We regret any confusion these errors may have caused. The article is corrected online, available at http://www.cdc.gov/ncidod/eid/vol8no1/01-0176.htm

